# The Arcuate Nucleus: A Site of Fast Negative Feedback for Corticosterone Secretion in Male Rats

**DOI:** 10.1523/ENEURO.0350-16.2017

**Published:** 2017-03-02

**Authors:** Luis Leon-Mercado, Daniela Herrera Moro Chao, María del Carmen Basualdo, Mitsuhiro Kawata, Carolina Escobar, Ruud M. Buijs

**Affiliations:** 1 Departamento De Biología Celular y Fisiología, Instituto De Investigaciones Biomédicas, Universidad Nacional Autónoma De México, 04510 Mexico City, Mexico; 2 Department of Medical Biochemistry, Academic Medical Center, 1105 AZ, Amsterdam, The Netherlands; 3 Department of Endocrinology and Metabolism, Academic Medical Center, 1105 AZ, Amsterdam, The Netherlands; 4 Department of Anatomy and Neurobiology, Kyoto Prefectural University of Medicine, Kyoto 602-8566, Japan; 5 School of Health Sciences, Bukkyo University, Kyoto 603-8301, Japan; 6 Departamento De Anatomía, Facultad De Medicina, Universidad Nacional Autónoma De México, 04510 Mexico City, Mexico

**Keywords:** ACTH, arcuate, glucocorticoid receptor, glucocorticoids, negative feedback, paraventricular nucleus

## Abstract

Variations in circulating corticosterone (Cort) are driven by the paraventricular nucleus of the hypothalamus (PVN), mainly via the sympathetic autonomic nervous system (ANS) directly stimulating Cort release from the adrenal gland and via corticotropin-releasing hormone targeting the adenohypophysis to release adrenocorticotropic hormone (ACTH). Cort feeds back through glucocorticoid receptors (GRs). Here we show in male Wistar rats that PVN neurons projecting to the adrenal gland do not express GRs, leaving the question of how the ANS in the PVN gets information about circulating Cort levels to control the adrenal. Since the arcuate nucleus (ARC) shows a less restrictive blood–brain barrier, expresses GRs, and projects to the PVN, we investigated whether the ARC can detect and produce fast adjustments of circulating Cort. In low Cort conditions (morning), local microdialysis in the ARC with type I GR antagonist produced a fast and sustained increase of Cort. This was not observed with a type II antagonist. At the circadian peak levels of Cort (afternoon), a type II GR antagonist, but not a type I antagonist, increased Cort levels but not ACTH levels. Antagonist infusions in the PVN did not modify circulating Cort levels, demonstrating the specificity of the ARC to give Cort negative feedback. Furthermore, type I and II GR agonists in the ARC prevented the increase of Cort after stress, demonstrating the role of the ARC as sensor to modulate Cort release. Our findings show that the ARC may be essential to sense blood levels of Cort and adapt Cort secretion depending on such conditions as stress or time of day.

## Significance Statement

Corticosterone secretion is importantly driven by preautonomic sympathetic neurons in the paraventricular nucleus of the hypothalamus (PVN), while negative feedback of glucocorticoids is generally expected to be mediated via the interaction of corticotropin-releasing hormone neurons in the PVN and corticotrophs in the hypophysis. Here, we demonstrate that the arcuate nucleus rapidly senses circulating corticosterone levels and thus adjusts the endocrine adrenal output via the preautonomic neurons in the PVN. This fast control may be an alternative mechanism used by circulating hormones and metabolites to communicate with control centers in the brain via the arcuate nucleus for fast feedback.

## Introduction

The control of glucocorticoids (GCs)—cortisol and corticosterone (Cort)—is known to be mediated by the endocrine portion of the paraventricular nucleus of the hypothalamus (PVN) modulating the secretion of adrenocorticotropic hormone (ACTH) from the pituitary via the release of corticotropin-releasing hormone (CRH) and vasopressin in the median eminence to the portal circulation (Kleitman and Holzwarth, 1985; Kalsbeek et al., 1992). However, rats maintained with constant circulating levels of ACTH after hypophysectomy still display a clear circadian peak of Cort before the activity period (Meier, 1976). In contrast, this peak is lost after denervation of the adrenal gland (Ottenweller et al., 1978; Jasper and Engeland, 1994), demonstrating the necessity of the autonomic input to the adrenal gland to release Cort in a circadian manner, probably due to modifications of sensitivity to ACTH in the adrenal gland (Jasper and Engeland, 1994; Ulrich-Lai et al., 2006; Lilley et al., 2012).

In the hypothalamus, Cort binds to the following two receptors: type I or mineralocorticoid receptor; and type II or glucocorticoid receptor (GR; Reul and De Kloet, 1985). When Cort binds to these receptors, the complex is quickly translocated from the cytoplasm to the nucleus in a dose- and time-dependent manner (Nishi et al., 1999). The type I receptor has 10 times more affinity for Cort and is rapidly saturated, while the type II receptor has lower affinity to Cort and is a high-capacity receptor. The type I receptor has been described as an important component of basal Cort homeostasis due to the high occupation under low-Cort situations, and the type II receptor is thought to respond mainly to high levels of Cort (Reul and De Kloet, 1985; Reul et al., 1987; de Kloet, 1991; Atkinson et al., 2008).

Cort regulation also requires a system of negative feedback to the CNS, which has been predominantly attributed to the cortex, amygdala, hippocampus, PVN, or the pituitary gland (Tasker, 2006), where the GR is highly expressed (Aronsson et al., 1988; Nishi et al., 2004). Although the pituitary gland expresses receptors to GCs, the fact that Cort can increase independently of appreciable modification of ACTH levels indicates the presence of additional regulatory mechanisms (Buijs et al., 1999; Ishida et al., 2005). Moreover, when GR is knocked down selectively in the PVN, the circadian release of Cort remains unaffected and the stress response is only slightly modified (Solomon et al., 2015). These results challenge the view of the PVN sensing GCs to give effective negative feedback.

An indication for alternative control mechanisms for Cort secretion came after polysynaptic retrograde neuronal tracing from the adrenal cortex, identifying first-order neurons in the intermediolateral column followed by neurons in the PVN, and third-order neurons in the arcuate nucleus (ARC). Consequently, we hypothesized that the identified projections from the ARC to PVN neurons projecting to the adrenal gland may serve to integrate information about circulating Cort levels to fine-tune endocrine function (Buijs et al., 2001).

In the ARC, type I and type II glucocorticoid receptors are highly expressed (Morimoto et al., 1996; Nishi et al., 2004; Han et al., 2005). ARC type II receptor concentration is one of the highest within the brain together with the hippocampus and PVN (Morimoto et al., 1996). In the ARC, two major subsets of neurons have been reported to be responsive to Cort with electrophysiological and transcriptional changes (Shimizu et al., 2008, 2010; Gyengesi et al., 2010), as follows: one coexpresses the orexigenic agouti-related peptide (AGRP) and neuropeptide Y (NPY), while α-melanocyte stimulating hormone, an anorexigenic peptide, is present in the other population together with the cocaine- and amphetamine-related transcript (Fan et al., 1997).

Here, we provide evidence that GR dispersion of GR after adrenalectomy is rapidly reversed in the ARC after intravenous Cort administration, whereas in other brain regions, including the PVN, it is reversed later. In addition, we illustrate the absence of GR in preautonomic neurons of the PVN projecting to the adrenal gland. This result, together with a specific response to GR antagonist in the ARC, but not in the PVN, points to the ARC as the site to control Cort release via the autonomic nervous system (ANS). Retrodialysis in the ARC with GR agonists and antagonists at different time points or after stress indeed established the role of the ARC as a sensor of hormonal fluctuations in the circulation to fine-tune Cort release under different physiologic conditions. This circuit may comprise projections from AGRP neurons to the preautonomic part of the PVN, which is connected by a polysynaptic pathway to the adrenal gland.

## Materials and Methods

### Animal handling

All animal experiments were performed in accordance with the regulations of the Universidad Nacional Autónoma de México and Instituto de Investigaciones Biomédicas, according to Mexican norms for animal handling (Norma Oficial Mexicana NOM-062-ZOO-1999) and the law for animal protection published in Mexico City, Mex. (February 2002) authors’ university animal care committee. Male Wistar rats weighing 250–350 g (Animal facility of the Faculty of Medicine, UNAM, animal facility of the Instituto de Investigaciones Biomédicas, UNAM) were housed in individual cages, with a 12 h light/dark schedule [lights on at 7:00 A.M., or zeitgeber time 0 (ZT0)]. The number of animals used was reduced according to ethical dispositions. Standard laboratory rodent diet (catalog #5013, LabDiet) and tap water were provided *ad libitum*. At the end of the protocols, all rats were deeply anesthetized with pentobarbital (100 mg/kg; Pisabental, Pisa Agropecuaria S.A. de C.V.) and perfused transacardially with 0.9% saline followed by a solution of 4% paraformaldehyde (PFA) in 0.1 m PB, pH 7.4. Brains were removed and kept in PFA 4% at 4°C for 24 h and subsequently changed to 30% sucrose with 0.02% sodium azide in 0.1 m PBS for cryoprotection until cut.

### Experimental design

To determine the rapid response of the GR to circulating Cort levels, rats were randomly assigned to one of the four following groups: intact (*n* = 6); adrenalectomy plus vehicle (*n* = 8); adrenalectomy plus Cort (*n* = 6); and adrenalectomy plus dexamethasone (Dex; *n* = 10). After 1 week of recovery, the animals were given intravenous sterile saline (100 µl), dexamethasone (100 µl, 0.05 mg/kg; Sigma-Aldrich), or corticosterone–HBC (2-hydroxypropyl-β-cyclodextrin) complex (100 µl of 0.5 mg/kg Cort; Sigma-Aldrich).

To determine the role of the ARC or PVN for GCs, negative-feedback rats were randomly assigned to one of the following groups: rats bearing bilateral cannulae in the PVN retrodialyzed with vehicle (*n* = 4) or type I GR antagonist (*n* = 4; eplerenone 160 µmol/L, Sigma-Aldrich); and rats bearing bilateral cannulae in the ARC retrodialyzed with vehicle (*n* = 4) or type I GR antagonist (*n* = 4; eplerenone 160 µmol/L, Sigma-Aldrich).

To determine the participation of type I and II GRs in the ARC for circadian regulation of Cort, rats bearing bilateral cannulae in the ARC were assigned to one of the following groups: vehicle (*n* = 4); type I GR agonist (*n* = 4; fludrocortisone 180 µmol/L, Sigma-Aldrich) or type I GR antagonist (*n* = 4; eplerenone 160 µmol/L, Sigma-Aldrich); and type II GR agonist (*n* = 5; dexamethasone, 200 µmol/L, Sigma-Aldrich) or type II GR antagonist (*n* = 4; mifepristone, 180 µmol/L, Sigma-Aldrich) infusion at ZT2. At ZT10, rats bearing bilateral cannulae in the ARC were assigned to treatment with vehicle (*n* = 4), type I GR agonist (*n* = 4), type I GR antagonist (*n* = 4), type II GR agonist (*n* = 5), or type II GR antagonist (*n* = 4).

To further explore the capacity of GCs to give negative feedback in the ARC, animals were exposed to mild stress. Animals bearing bilateral cannulae in the ARC were assigned randomly to the following groups: no stress plus vehicle (*n* = 4); no stress plus Dex (*n* = 5); no stress plus fludrocortisone (*n* = 4); stress plus vehicle (*n* = 4); stress plus Dex (*n* = 4); and stress plus fludrocortisone (*n* = 4).

### Transneuronal tracing

Animals were anesthetized with sodium pentobarbital (50 mg/kg). Three microliters of the viral suspension of pseudorabies virus (PRV), Bartha strain (containing 1 × 10^6^ plaque-forming units), was pressure injected into the left adrenal gland with a 30 gauge needle attached to a Hamilton syringe. Evans blue was added to the viral suspension to allow visual inspection of the injection site. Animals survived for 3–4 d.

### Adrenalectomy

Animals underwent surgeries according to the different experimental designs under anesthesia with intramuscular ketamine (40–80 mg/kg; Anesket, Pisa Agropecuaria S.A. de C.V.) and intraperitoneal xylazine (5–8 mg/kg; Procin, Pisa Agropecuaria S.A. de C.V.). The adrenal glands were removed bilaterally by a cut on the back of the animal, and silicon catheters (SILASTIC) were inserted into the right jugular vein. After 1 week of recovery, the animals were given intravenous sterile saline (100 µl), dexamethasone (0.05 mg/kg; Sigma-Aldrich), or corticosterone–HBC complex (0.5 mg/kg Cort; Sigma-Aldrich). The animals were killed 7 min after the injection, as described previously.

### Cannulation and stereotactic surgery for retrodialysis

Animals were anesthetized with ketamine and xylazine, as described previously. Silicon catheters (SILASTIC) were inserted into the right jugular vein for blood sampling. Homemade microdialysis probes were placed bilaterally into the ARC or the PVN with a standard Kopf stereotaxic apparatus. Coordinates were adapted from the atlas of Paxinos and Watson (Paxinos and Watson, 2013) (2007; PVN: anteroposterior, −1.1 mm; lateral, +0.9 mm; ventral, −6.9 mm; tooth bar, −2.5°; lateral angle, 4°; ARC: anteroposterior, −2.4 mm; lateral, +1.7 mm; ventral, −9.5 mm; tooth bar, −3.4°). The catheter and microdialysis probes were fixed on top of the skull with stainless steel screws and secured with dental acrylic. The permeability of the jugular catheter was maintained with a solution of 45% glycerol, 40% isotonic saline, 10% heparin (5000 IU/ml; Inhepar, Pisa Agropecuaria S.A. de C.V.), and 5% Antibiotic Mixture 100× (Gibco) replaced every 2 d.

### Retrodialysis

For bilateral retrodialysis, the probes were placed into the PVN or ARC. The positions of the cannulae were verified at the end of the protocol by histologic examination with Nissl staining. Animals with misplaced probes were excluded from analysis. After 1 week of recovery, animals were connected to a multichannel fluid infusion swivel (Instech Laboratories) 1 d before the experiment for adaption to prevent any effect of stress on the day of the experiment.

#### Morning experiments

On the day of the experiment, the animals with a bilateral probe in the ARC received retrodialysis with Ringer’s solution (3 µl/min) from ZT0 to ZT2. Next, the ringer solution of retrodialysis was replaced by the corresponding drugs at the following concentrations at the same infusion rate: vehicle; type II GR agonist (dexamethasone 200 µmol/L, Sigma-Aldrich); type II GR antagonist (mifepristone 180 µmol/L, Sigma-Aldrich); type I GR agonist (fludrocortisone 180 µmol/L, Sigma-Aldrich); or type I GR antagonist (eplerenone 160 µmol/L, Sigma-Aldrich). Two hundred microliters of blood were taken 5 min before and 5, 10, 20, 40, and 80 min after starting the drug retrodialysis. Drugs were dissolved in EtOH 0.5% in Ringer’s solution, which was used as the vehicle solution.

Animals with PVN bilateral probes received retrodialysis with Ringer’s solution followed by vehicle or eplerenone, as described previously.

#### Afternoon experiments

The day of the experiment, the animals received retrodialysis with Ringer’s solution from ZT8 to ZT10. Afterward, the Ringer’s solution was replaced by the corresponding drugs, and blood samples were taken as described previously.

#### Stress experiments

Animals received retrodialysis with Ringer’s solution (3 µl/min) from ZT0 to ZT2. Subsequently, stress was induced by transferring the animal to a new clean home cage, just before the change of drug infusion by retrodialysis. Animals were assigned randomly to the following groups: no stress plus vehicle; no stress plus Dex; no stress plus fludrocortisone; stress plus vehicle; stress plus Dex; and stress plus fludrocortisone.

### Immunohistochemistry

Brains were cut into 40 µm coronal sections with a cryostat (catalog #HM550, Microm) and stored in 30% sucrose with 0.02% sodium azide in 0.1 m PBS until staining. Sections for immunolabeling (Six per region per animal) were collected and rinsed in 0.1 m PBS, and then were incubated for 10 min with hydrogen peroxide 3% in 0.1 PBS, followed by overnight incubation with blocking solution (0.1% fraction V bovine albumin, 0.2% triton X-100 in 0.1 m PBS). All the antibodies were diluted in blocking solution. The primary antibodies were used as follows: rabbit anti-PRV (1:150,000; antialpha Aujerszky, a generous donation by Dr. H. Pol, Dutch Institute for Animal Health and Science, Lelystad, The Netherlands), rabbit anti-GR (1: 16,000; homemade; Morimoto et al., 1996). The primary antibody was incubated for 1 h at room temperature (RT) and then 24–48 h under constant shaking at 4°C; afterward, sections were rinsed with washing buffer and incubated for 1 h at room temperature with the corresponding biotinylated secondary antibody (donkey anti-rabbit 1:1000, Jackson ImmunoResearch; donkey anti-goat 1:1000, Jackson ImmunoResearch), rinsed, and incubated in avidin–biotin complex (1:500; Vector Laboratories) for 1 h. Product visualization was obtained with 0.01% diaminobenzidine (DAB), 0.05% nickel ammonium sulfate, and 0.01% hydrogen peroxide in TBS 0.1 m for 8–10 min. When a second staining was performed, the same sections were incubated, as described previously, with primary antibody followed by incubation with biotinylated secondary antibody and afterward incubated in avidin–biotin complex. Product visualization was obtained with 0.01% DAB and 0.01% hydrogen peroxide in TBS 0.1 m for 5–8 min. The sections were rinsed and changed to a solution of gelatin 0.25% and 0.04% sodium azide in PBS 0.1 m and placed on gelatinized glass slides. After drying, the sections were dehydrated with alcohol and xylene and coverslipped with Entellan (Merck).

For fluorescent immunohistochemistry, after incubation with the primary antibody, sections were incubated for 1 h with donkey anti-rabbit Cy2 (1:500; Jackson ImmunoResearch), donkey anti-goat (1:500; Jackson ImmunoResearch), or donkey anti-sheep (1:500; Jackson ImmunoResearch) as convenient, rinsed with washing buffer, and mounted in gelatin-coated slides. Finally, slides were coverslipped with Mowiol mounting medium. Bright-field photomicrographs were taken with a digital camera (Infinity 2-5 URFC color camera, Lumenera) and Infinity Capture and Analyze software, and fluorescent photomicrographs were acquired with a confocal microscope (A1R^+^ STORM, Nikon).

### *In situ* hybridization

#### Probes

The sequences used for AGRP were as follows: forward primer, TCCCAGAGTTCTCAGGTCTAAGTC (Sigma-Aldrich), and reverse primer, ACAGCGACGCGGAGAACGAGA (Sigma-Aldrich), with product length of 134 bp. The sequences used for POMC were as follows: forward primer, AGGTGTACCCCAATGTTCGC (Sigma-Aldrich), and reverse primer, ACCCTCACTGGCCCTTCTTG (Sigma-Aldrich), with a final product length of 476 bp. The reverse primers were labeled in 5' with T7 polymerase promoter.

DIG-RNA labeling was obtained using 200 ng of purified primer, 2 µl of DIG Labeling Mix (Roche), 2 µl of T7 RNA polymerase (Roche), 2 µl of 10× concentrated transcription buffer (Roche), 40 U of RNase out (Invitrogen), and Milli-Q DEPC water to reach 20 µm of total reaction volume. The mix was incubated for 2 h at 37°C in water bath. To improve the labeling reaction, 1 µm of RNA polymerase was added to the reaction mixture and was incubated for 1 h at 37°C. Afterward, 2 µl of 0.2 m EDTA, pH 8.0, 2.5 µl of 4 m LiCl, and 75 µl of prechilled (in a −20°C freezer) 100% ethanol were added to the reaction mix and incubated overnight at −20°C. Fifty microliters of prechilled ethanol were added, the mix was centrifuged for 10 min at 14,000 rpm at 4°C, the supernatant was removed, and the tube was left open in ice to dry for 10 min. The samples were resuspended in 100 µl of sterile RNase-free water.

The DIG-labeling reaction efficiency was determined by spot assay. The RNA dilutions were spotted on Nylon Membrane (Zeta Probe, Bio-Rad) incubated with anti-DIG POD (Roche), and revealed with DAB. The color intensity was compared between labeled control RNA (Roche) and problem samples. The optimal probe concentration was confirmed in brain slices.

#### Hybridization

Brains were removed and kept in PFA 4% at 4°C for 24 h and subsequently changed to a solution of 30% sucrose RNase free with 0.02% sodium azide in 0.1 m PBS for cryoprotection until cut. Brains were cut in 40 µm coronal sections with a cryostat (catalog #HM550, Microm) and stored in 0.1 m PBS until staining.

Sections (six per region per animal) were collected and rinsed for 10 min in 0.1 m PBS, then fixed in 4% PFA in PBS for 15 min and rinsed with Triton X-100 0.03% in PBS RNase free (washing buffer). Afterward, the sections were treated for 30 min with active DEPC 0.1% in 0.1 m PBS, rinsed with washing buffer and incubated for 30 min with hydrogen peroxide 3% in 0.1 PBS, rinsed with washing buffer and incubated for 10 min at RT with 0.25% acetic anhydride in 0.1 m 0.1 m triethanolamine/Milli-Q DEPC water, rinsed and incubated for 1 h at 63°C with hybridization solution without probe (4× SSC, 50% deionized formamide, 1× Denhardt’s solution, 0.02% Ficoll, 0.02% polyvinylpyrolidone, and 0.02% bovine serum albumin) in a hybridization oven (model 230402, Boekel Scientific). The sections were then incubated overnight at 63°C with RNA probes previously denatured in hybridization solution for 5 min at 85°C in water bath followed by ice immersion for 5 min. After incubation, the sections were washed with 2× SSC without formamide at RT, and successive washings with 5× SSC with 50% formamide, 2× SSC with 50% formamide, 0.2× SSC 50% formamide for 15 min at 63°C, and finally 2× SSC for 5 min at RT. Subsequently, the tissue was incubated in buffer 1 (100 ml of Tris HCl 1 m, pH 7.4, 30 ml of NaCl 5 m, and 870 ml of Milli-Q water treated with DEPC) for 5 min and incubated with blocking solution 1% (Roche) in buffer 1 for 30 min. Next, the sections were incubated with sheep anti-DIG-POD antibody (1:1000, Roche) in buffer 1 with blocking solution for 2 h at RT. Sections were rinsed with washing buffer, and the signal was amplified with TSA Plus Biotin Kit (PerkinElmer), as indicated by the provider. Finally, the sections were incubated with streptavidin AP for 1 h at RT (1:000; Jackson ImmunoResearch). Afterward, the sections were rinsed with washing buffer, and the color was revealed with liquid Fast-Red substrate kit (Abcam). The incubation time was ∼20 min. Immunohistochemistry was performed after hybridization, as described previously.

### Cell counts

For the quantification of the GR immunoreactivity (IR)-positive cells, two anterior, two medial, and two posterior sections of the cingulate cortex, central and medial amygdala, dentate gyrus of the hippocampus, PVN, and ARC were selected based on a stereotactic atlas. Images of selected sections were digitalized at 20× magnification using computerized images were obtained with a digital camera (Infinity 2-5 URFC Color Camera, Lumenera) and Infinity Capture and Analyze software, and the number of GR-positive profiles was selected using ImageJ (National Institutes of Health) bilaterally for the intact, adrenalectomy plus vehicle, adrenalectomy plus Dex, and adrenalectomy plus Cort groups.

### Analytical methods

Blood samples were kept in ice and centrifuged at 4000 rpm for 8 min. The serum was collected and stored at −20°C. Serum corticosterone concentrations were measured using RIA kits (MP Biomedical), serum ACTH levels were measured with an enzyme immunoassay kit (Phoenix Pharmaceuticals) and aldosterone with ELISA (Alpco), as indicated by the providers.

### Statistical analysis

Values are shown as the mean ± SEM for each group. Comparisons were performed using two-way repeated-measures (RM) ANOVA, followed by Tukey’s multiple-comparison test (INStat; Prism 6.01 Software, GraphPad). The association between variables was explored by simple linear regression. *p* Values <0.05 indicate a statistical significant difference. Table 1 contains more information regarding the statistical results presented.

**Table 1:** Data distribution and statistical results. Normality tested using Kolmogorov–Smirnov test with Dallal–Wikinson–Lille test for *p* value

Figure	Panel	Data structure	Test type	*p* value
1	*P*	Normally distributed	One-way ANOVA	<0.0001
1	*Q*	Normally distributed	One-way ANOVA	<0.0001
1	*R*	Normally distributed	One-way ANOVA	<0.0001
1	*S*	Normally distributed	One-way ANOVA	<0.0001
1	*T*	Normally distributed	One-way ANOVA	<0.0001
3	*A*	Normally distributed	Two-way RM ANOVA	0.0002
4	*A*	Normally distributed	Two-way RM ANOVA	0.0012
4	*B*	Normally distributed	Two-way RM ANOVA	0.3361
4	*C*	Normally distributed	Two-way RM ANOVA	0.0297
4	*D*	Normally distributed	Two-way RM ANOVA	0.7184
5	*A*	Normally distributed	Two-way RM ANOVA	0.2709
5	*B*	Normally distributed	Two-way RM ANOVA	0.4094
5	*C*	Normally distributed	Two-way RM ANOVA	0.0012
5	*D*	Normally distributed	Two-way RM ANOVA	0.0106
6	*A*	Normally distributed	Two-way RM ANOVA	0.4819
6	*B*	Normally distributed	Two-way RM ANOVA	0.8865
7	*A* Vehicle ZT2	Normally distributed	Linear regression	0.0741
7	*B* Dexamethasone ZT2	Normally distributed	Linear regression	0.6525
7	*A* Fludrocortisone ZT2	Normally distributed	Linear regression	0.4217
7	*B* Mifepristone ZT2	Normally distributed	Linear regression	0.6743
7	*A* Eplerenone ZT2	Normally distributed	Linear regression	0.0318
7	*C* Vehicle ZT10	Normally distributed	Linear regression	0.1518
7	*D* Dexamethasone ZT10	Normally distributed	Linear regression	0.242
7	*C* Fludrocortisone ZT10	Normally distributed	Linear regression	0.504
7	*D* Mifepristone ZT10	Normally distributed	Linear regression	0.4581
7	*C* Eplerenone ZT10	Normally distributed	Linear regression	0.9576
8	*A*	Normally distributed	Two-way RM ANOVA	0.0005
8	*B*	Normally distributed	Two-way RM ANOVA	0.003

## Results

### The GRs in the ARC rapidly respond to circulating Cort levels

**Figure 1.**
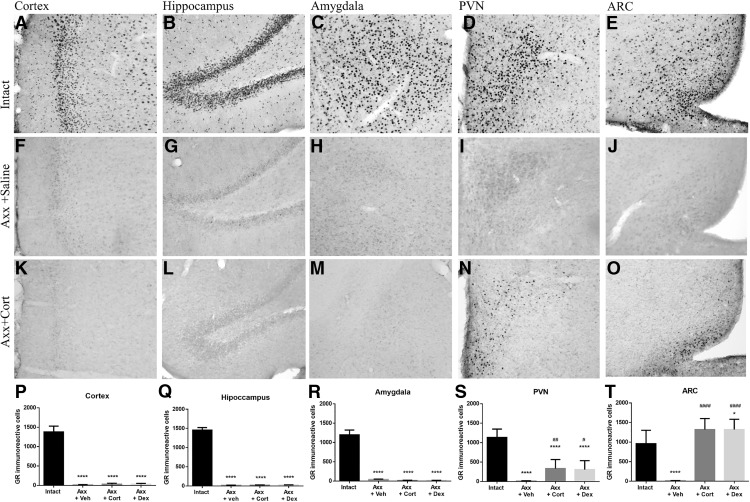
***A–J***, GR IR is abundant in intact animals (***A–E***; *n* = 6), blunted after adrenalectomy (***F–J***), and selectively recovered after Dex or Cort administration. ***K–M***, ***P–R***, Seven minutes after injection of vehicle (*n* = 6), Cort (*n* = 6), or Dex (*n* = 10) into the circulation of adrenalectomized animals, GR is not recovered in the cortex (***K***, ***P***), hippocampus (***L***, ***Q***), or amygdala (***M***, ***R***). ***N***, ***O***, ***S***, ***T***, In contrast, intravenous injection of Cort or Dex partly recovers GR IR in the PVN (***N***, ***S***) and the ARC GR IR is fully recovered shortly after administration of Cort and Dex (***O***, ***T***). Asterisks indicate statistically significant differences from the intact group: *p < 0.05; **p < 0.01; ***p < 0.001; ****p < 0.0001. Hash symbols indicate significant differences from the adrenalectomized plus vehicle group: #*p* < 0.05; ##*p* < 0.01; ###*p* < 0.001; ####*p* < 0.0001. All data are presented as the mean ± SEM. Scale bar, 100 µm.

In intact animals treated with vehicle intravenously, GR IR is ubiquitously present in the brain (e.g., cortex, hippocampus, amygdala, PVN, and ARC; Fig. 1*A–E*). As GR nuclear IR is lost in the absence of Cort and is rapidly recovered within minutes after agonists bind to GR (Nishi et al., 1999), we decided to use the reappearance of GR IR 7 min after intravenous administration of Cort or Dex to investigate the speed of access of circulating Cort. Adrenalectomy caused a complete loss of GR IR in the brain, as reported previously (Hu et al., 1999), and intravenous administration of saline, Cort, or Dex did not produce GR IR in the cortex (*F* = 116.1; *p* < 0.0001), amygdala (*F* = 120.9; *p* < 0.0001), and hippocampus (*F* = 599.6; *p* = 0.0001; Fig. 1*F–H*,*K–M*). GR IR-positive cells decreased after adrenalectomy, and GR IR was recovered especially in the ventromedial ARC (*F* = 49.76; *p* < 0.0001) and to some extent in the PVN (*F* = 40.03; *p* < 0.0001) with no differences between Dex or Cort (Fig. 1*I*,*J*,*N–T*) 7 min after intravenous Cort or Dex administration, but not earlier. GR IR was recovered ubiquitously in the brain 50 min after the administration of Dex (data not shown).

**Figure 2.**
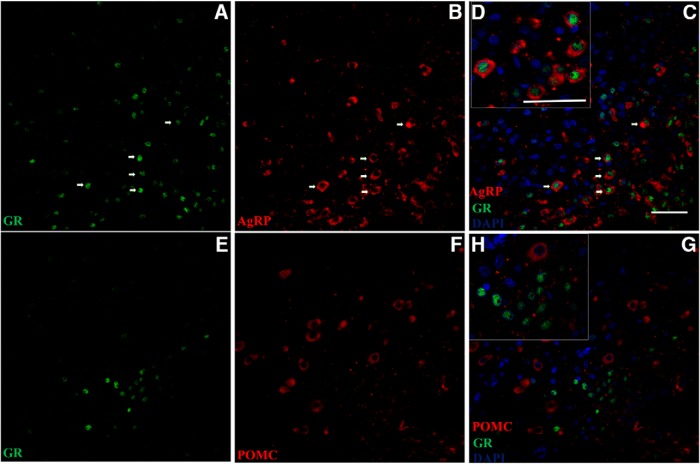
***A–C***, ***E–G***, Photomicrographs showing the recovery of GR in AGRP neurons (***A–C***) but not in POMC neurons 7 min after Cort injection into the circulation (***E–G***). Scale bar, 50 µm. ***D***, ***H***, Higher magnification of AGRP (***D***) and POMC (***H***) sections. Scale bar, 50 µm. White arrows indicate neurons with GR and AGRP or POMC. DAPI, blue; GR, green; POMC or AGRP, red.

Within the ARC, GR-positive cells were distributed mainly in the region close to the third ventricle, the classic AGRP/NPY neuronal location. Subsequently, since GR was not recovered homogenously in the ARC, we characterized the neurons responsive to circulating Cort by means of *in situ* hybridization for AGRP or POMC combined with GR immunohistochemistry. We observed that GR-positive neurons were also positive for AGRP (Fig. 2*A–D*), but not for POMC (Fig. 2*F–I*).

These results illustrate the privileged capacity of the ARC to rapidly sense circulating molecules, either via the fenestrations of the blood–brain barrier in the case of ARC or later for the PVN via the CSF, once these molecules have entered the third ventricle via the ARC–median eminence complex. This phenomenon has been previously reported for having no steroid hormones, such as ghrelin and leptin (Schaeffer et al., 2012; Balland et al., 2014; Djogo et al., 2016).

### ARC, but not PVN, gives negative feedback to GCs

**Figure 3.**
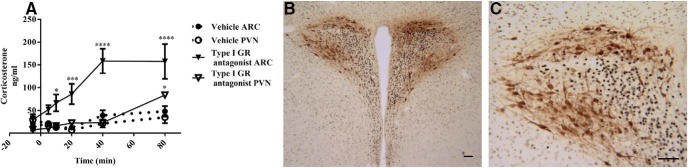
ARC, but not PVN, provides negative feedback to GC release from adrenal glands. ***A***, Plasma concentration of Cort during retrodialysis in the ARC (closed figures) or PVN (open figures) with vehicle (dotted lines: ARC, *n* = 4; PVN, *n* = 4) or eplerenone (continuous lines: ARC, *n* = 4; PVN, *n* = 4). ***B***, Photomicrographs of the PVN for identification of GR-positive cells (black nuclear staining) and PRV-positive cells (brown, cytosolic staining) demonstrating the absence of GR in PVN neurons projecting multisynaptically to the adrenal gland (*n* = 6). Scale bar, 100 µm. ***C***, Higher magnification of PVN (Scale bar = 100 µm). Asterisks indicate statistically significant differences: *p < 0.05; **p < 0.01; ***p < 0.001; ****p < 0.0001. All data are presented as the mean ± SEM.

Based on the recovery of GR IR in both the ARC and PVN shortly after intravenous administration of Cort, we investigated their capacity to produce negative feedback on Cort. We compared the effects of reverse microdialysis with type I GR antagonist (eplerenone) either in the ARC or PVN and obtained blood samples at 5 min before and 10, 20, 40, and 80 min during the infusion. The RM ANOVA pointed to differences in time (*F*_(5,60)_ = 20.17; *p* < 0.0001) and place of drug infusion (*F*_(3,12)_ = 16.43; *p* = 0.0002). Infusion in the ARC caused an increase in Cort at 10 min and continued to the end of the infusion. In contrast, retrodialysis in the PVN caused an increase of Cort only after 80 min (Fig. 3*A*).

Since type I antagonist infusion in the PVN produced a late change in Cort production, we investigated whether both preautonomic and endocrine portions in the PVN express GR, given that both neuronal populations are described to contribute to GC production. Following inoculation of PRV in the cortex of the adrenal gland, we identified neurons positive for GR and those positive for PRV by double immunohistochemistry. We observed two separate populations, with the highest amount of GR present in the medial parvocellular part of the PVN and absent in the preautonomic PVN neurons polysynaptically connected to the adrenal gland (Fig. 3*B*,*C*).

Together, these results suggest that circulating Cort reaches rapidly GR-positive neurons in the ARC to quickly increase circulating Cort levels. This fast negative feedback seems to involve ARC–AGRP projections to preautonomic neurons in the PVN. The delayed response of the PVN to the GR antagonist seems to be due to the absence of GR in the preautonomic neurons projecting to the adrenal gland.

### Occupancy of type I and type II GR in the ARC gives differential negative feedback during circadian trough and peak of Cort

Since sympathetic input to the adrenal gland is necessary to induce an adequate circadian rhythm of Cort, we hypothesized that the ARC could participate in the circadian regulation of corticosterone Cort release transmitting the signal about circulating Cort to the autonomic portion of the PVN.

GCs act via two different receptors along the circadian cycle. Previous studies have demonstrated that the type I receptor, and not type II, is occupied with low concentrations of Cort, while the type II receptor is occupied when the Cort level is higher (Reul et al., 1987) . We therefore tested the pharmacological agonist for type I or type II GR (fludrocortisone and dexamethasone, respectively) or their antagonists (mifepristone and eplerenone) under unstressed conditions in the morning (ZT2) or afternoon (ZT10) with retrodialysis in the ARC.

**Figure 4.**
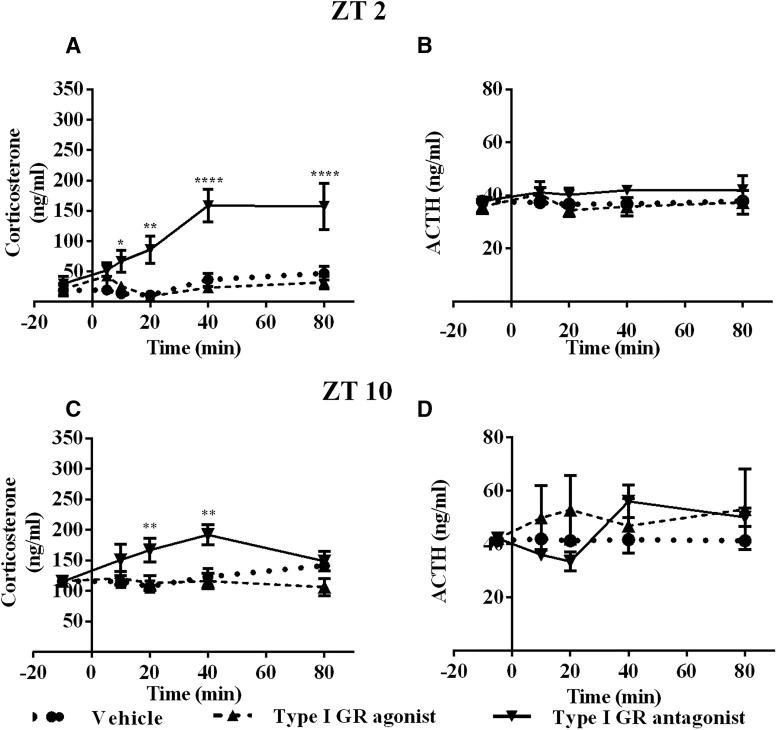
Differential involvement of type I receptor during the circadian trough and peak of Cort. ***A***, ***B***, Plasma concentration of Cort (***A***) and ACTH (***B***) during retrodialysis with vehicle (dotted line, *n* = 4), agonist (dashed line line, *n* = 4), or antagonist (continuous line *n* = 4) to type I receptor At ZT2. ***C***, ***D***, Plasma concentration of Cort (***C***) and ACTH (***D***) during retrodialysis with vehicle (dotted line, *n* = 4), agonist (dashed line, *n* = 4), or antagonist (dotted line *n* = 4) to type I receptor At ZT10 (agonist *n* = 5, antagonist *n* = 4). Asterisks indicate significant differences: *p < 0.05; **p < 0.01; ***p < 0.001; ****p < 0.0001. All data are presented as the mean ± SEM.

**Figure 5.**
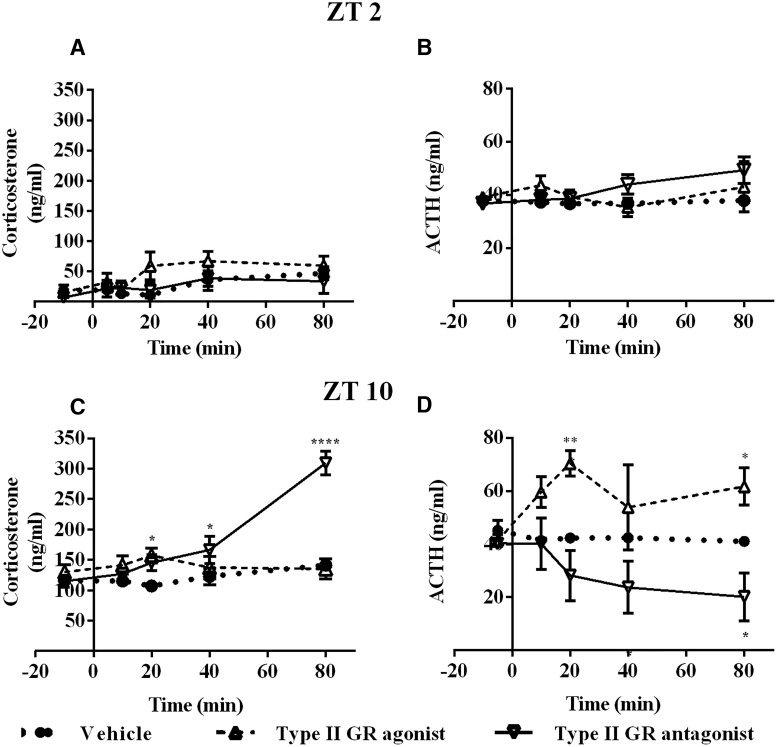
Differential involvement of type II receptor during the circadian trough and peak of Cort. ***A***, ***B***, Plasma concentration of Cort (***A***) and ACTH (***B***) during retrodialysis with vehicle (dotted line, *n* = 4), type II receptor agonist (dashed line, *n* = 5), or antagonist (continuous line *n* = 4) At ZT2. ***C***, ***D***, Plasma concentration of Cort (***C***) and ACTH (***D***) during retrodialysis with vehicle (dotted line, *n* = 4), agonist (dashed line, *n* = 4), or antagonist (continuous line *n* = 4) at ZT10. Asterisks indicate significant differences: *p < 0.05; **p < 0.01; ***p < 0.001; ****p < 0.0001. All data are presented as the mean ± SEM.

In the morning, at ZT2, retrodialysis with type I GR agonist did not change Cort, while type I GR antagonist produced a fast increase in Cort compared with the vehicle (*F*_(2,9)_ = 15.67; *p* = 0.0012; Fig. 4*A*). In contrast, neither type II GR antagonist or agonist produced any effect on Cort when compared with vehicle infusion (*F*_(2,10)_ =1.493; *p* = 0.279; Fig. 5*A*). Retrodialysis with type I receptor agonist or antagonist did not cause changes in ACTH (*F*_(2,9)_ = 1.234; *p* = 0.3361; Fig. 4*B*). Retrodialysis with type II receptor agonist or antagonist did not cause changes in ACTH (*F*_(2,9)_ = 0.9880; *p* = 0.4094; Fig. 5*B*).

**Figure 6.**
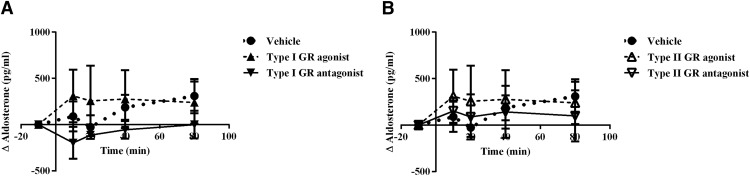
Aldosterone levels do not change by GCs in the ARC. ***A***, ***B***, Plasma concentration of aldosterone during retrodialysis with agonist (dashed line) or antagonist (dotted line) to type I receptor (***A***) and type II receptor (***B***) at ZT2. All data are presented as the mean ± SEM.

Taking into account previous reports suggesting that the increase of other corticosteroids after sympathetic stimulation or increased ACTH (Keller-Wood et al., 1986; Bornstein et al., 1990; El Ghorayeb et al., 2016), we measured aldosterone during the retrodialysis. We did not find any significant variation in aldosterone production after stimulation or inhibition of type I GR (*F*_(2,6)_ = 0.8265; *p* = 0.4819) or type II GR (*F*_(2,6)_ = 0.1229; *p* = 0.8865) in the ARC, indicating that the induced adrenal stimulation is specific for Cort (Fig. 6*A*,*B*) despite the use of type I receptor agonist and antagonist.

At ZT10 (late afternoon), when Cort nearly reaches its daily peak (Kalsbeek et al., 1996), the change in Cort levels in the group treated with the type I receptor antagonist differed from the those in the vehicle group at 20 and 40 min after the beginning of the treatment and were similar to the Cort levels of the vehicle-treated group at 80 min (*F*_(2,11)_ = 4.925; *p* = 0.0297; Fig. 4*C*). In contrast to ZT2, the group infused with type II GR antagonist showed a steady increase in Cort levels immediately after beginning of the treatment (*F*_(2,10)_ = 14.11; *p* = 0.0012; Fig. 5*C*). Type I GR agonist or antagonist in the ARC did not change the blood concentration of ACTH (*F*_(2,8)_ = 0.3448; *p* = 0.7184; Fig. 4*D*), while type II agonist and antagonist induced significant changes in circulating ACTH (*F*_(2,7)_ = 9.343; *p* = 0.0106; Fig. 5*D*), interestingly, these changes were not paralleled by Cort (Fig. 5*C*).

**Figure 7.**
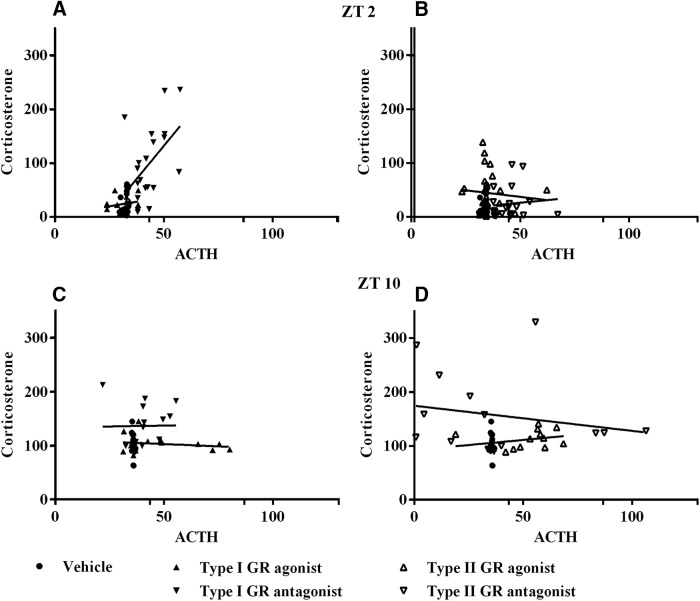
***A–D***, Linear regression of ACTH and Cort in plasma during dialysis with type I GR and type II GR agonist or antagonists at ZT2 (***A***, ***B***) and ZT10 (***C***, ***D***), illustrating the absence of correlation between ACTH and Cort levels.

Simple linear regression shows that there is no relationship between ACTH and Cort at ZT2 for vehicle (*p* = 0.0742; *R* ^2^ = 0.1665), type I GR agonist (*p* = 0.4217; *R* ^2^ = 0.05443), type I GR antagonist (*p* = 0.0318; *R* ^2^ = 0.2312), type II GR agonist (*p* = 0.06525; *R* ^2^ = 0.0089), or type II GR antagonist (*p* = 0.6743; *R* ^2^ = 0.01004; Fig 7*A*,*B*). In the afternoon, dialysis with vehicle (*p* = 0.1518; *R* ^2^ = 0.1513), type I GR agonist (*p* = 0.5040; *R* ^2^ = 0.0350), type I GR antagonist, type II GR agonist, and type II GR antagonist did not show any correlation between ACTH and Cort (Fig. 7*C*,*D*). These results suggest that Cort release is mediated by the autonomic nervous system rather than via ACTH. Moreover, the present results provide physiologic evidence of the preferential occupation of type I receptor in the morning and the occupation of type II in the afternoon and demonstrate for the first time the importance of GR occupation in the ARC to regulate Cort. In addition, the contrast between circulating levels of ACTH and Cort, as observed with type II GR antagonist at ZT10, supports the autonomic control of Cort release.

### GR agonist in the ARC blunts the stress response

**Figure 8.**
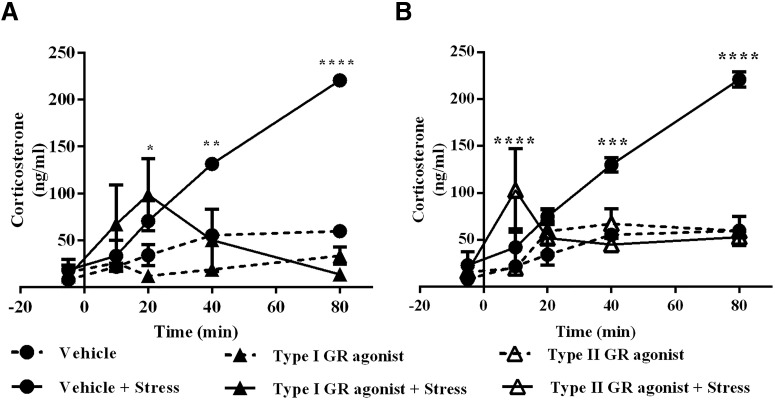
GR agonists in the ARC blunt the stress response. ***A***, ***B***, Plasma concentration of Cort during retrodialysis with agonist to type I receptor ▲ (***A***, *n* = 4), type II receptor Δ (***B***, *n* = 5), or vehicle ● (*n* = 4). Samples were obtained at ZT2 in control animals (dotted line) or stressed animals (continuous line: vehicle, *n* = 4; type I agonist, *n* = 4; type II agonist, *n* = 5). Asterisks indicate significant differences: *p < 0.05; **p < 0.01; ***p < 0.001; ****p < 0.0001. All data are presented as the mean ± SEM.

To further explore the capacity of GCs to give negative feedback in the ARC, we compared Cort release of unstressed animals with the release after exposing the animals to a novel environment as stressor at ZT2, when Cort levels are normally low (Buijs et al., 1997). Simultaneously with the stress, we administered vehicle, type I receptor agonist, or type II receptor agonist in the ARC by means of retrodialysis. The treatment with vehicle or agonists alone did not increase circulating levels of Cort. As expected, the animals exposed to stress and treated with vehicle displayed a rapid increase in circulating Cort levels, while the group treated with type I GR agonist after stress displayed only a peak at 20 min and returned to values comparable to vehicle-treated animals (*F*_(3,12)_ = 12.59; *p* = 0.0005; Fig. 8*A*). Similarly, the group treated with type II GR agonist after stress showed only a small increase in Cort levels at 10 min and reached vehicle values in later samples (*F*_(3,13)_ = 9.461; *p* = 0.0014; Fig 8*B*). These results further demonstrate the roles of type I and type II GRs in the ARC to monitor circulating Cort levels under diverse conditions providing fast negative feedback, as demonstrated in this experiment, preventing the expected elevation of Cort levels.

## Discussion

Since the discovery that Cort secretion is in large part regulated by the autonomic output of the PVN, studies were missing that would answer how the negative feedback for this arm of the PVN could take place. The present study adds to the negative feedback of Cort evidence of a fast feedback via neurons in the ARC. Here, low and high levels of Cort occupy, respectively, type I and type II GRs and do not change Cort levels via ACTH. Consequently, this information is transmitted polysynaptically to the adrenals. Present results confirm for the first time the early hypothesis that in the beginning of the rest period (ZT2), when circulating levels of Cort are low, only the type I GRs are occupied and contribute to the negative feedback of blood Cort levels. At the end of the rest period (ZT10), when Cort levels rise and the type II GRs are occupied, the type I GRs do not play a role anymore in the feedback, which is then taken over by the type II GRs (Reul and De Kloet, 1985; Reul et al., 1987; Evanson et al., 2010).

A few minutes after intravenous administration of Cort in adrenalectomized rats, the staining for GRs is recovered mainly in ARC AGRP neurons, indicating that most probably circulating Cort penetrates immediately via fenestrated capillaries in the ARC–median eminence complex (Fig. 1). The delay in GR staining to 7 min is most probably due to the delay in translocation of GR from the cytoplasm to the nucleus (Nishi et al., 1999). The delay in the appearance of GR staining in the cortex and hippocampus agrees with previous studies showing a delay of ∼20 min between the increase of Cort in the circulation and inside the hippocampus after stress (Droste et al., 2008). This observation demonstrates that brain structures other than the ARC are affected later by the changes of Cort. The immediate appearance of GR in AGRP neurons, but not POMC neurons (Fig. 2), agrees with previous observations of changing levels of AGRP mRNA in the ARC during the day mediated by Cort (Xin-Yun et al., 2002), by the action of type II GRs (White et al., 1994), by AMP-activated protein kinase (Shimizu et al., 2008), and by the identification of a brain-specific GR homeobox factor to regulate AGRP (Lee et al., 2013). Considering this and the projections of AGRP going from ARC to PVN (Baker and Herkenham, 1995) suggests that AGRP cells may transmit information related to GC status from the circulation to other hypothalamic structures such as the PVN.

The absence of response after GR antagonist infusion in the PVN, together with the lack of GR in preautonomic neurons (Fig. 3) indicates that Cort in the PVN is not enough to give fast negative feedback to GCs. This is supported by observations that animals with specific knockout of GR in the PVN still present similar levels of Cort before the activity period and under stress (Solomon et al., 2015), confirming that PVN neurons mediating GC release in a circadian manner do not express GR. The preautonomic portion of the PVN, although devoid of GR, can stimulate glucocorticoid release independently of the amount of ACTH via the activity of the splanchnic nerve (Buijs et al., 1993, 1999b; Ulrich-Lai et al., 2006). Thus, as we showed here, AGRP input from the ARC to the preautonomic portion of the PVN may transmit GC state to control splanchnic nerve tone and ACTH sensitivity in the adrenal gland.

Evidence for specific effects of microdialysis probe placement comes from the experiments comparing the effect on the PVN or on the ARC, since both hypothalamic nuclei are close enough to allow some effect of diffusion. However, our data clearly show that only cannulae placed correctly in the arcuate nucleus provide negative feedback when the drug is applied (Fig. 3). In addition, we have observed that misplaced cannulae outside the ARC (mostly in the ventromedial hypothalamic nucleus) did not have any effect on the release of Cort (data not shown).

The absence of a relationship between ACTH and Cort levels as presently observed has also been reported previously in several conditions as restricted feeding schedules or disruption of the resting/active phases via light exposure (Ishida et al., 2005; Buijs and Escobar, 2007). In the evening, the adrenal gland is more responsive to ACTH than in the morning, despite similar levels of circulating ACTH levels (Dallman et al., 1978; Kaneko et al., 1981). In the same sense, experiments conducted in suprachiasmatic nucleus-lesioned animals or *in vitro* did not show this rhythm (West and Bassett, 1992; Buijs et al., 1993). Together, these observations can be interpreted as differences in adrenal responsiveness to ACTH mediated by the autonomic output to the adrenal cortex. This could be confirmed in additional experiments manipulating the GR in the ARC of hypophysectomized animals maintained with constant levels of ACTH.

We show that the use of agonists does not change significantly the amount of ACTH released during the dialysis at any condition at ZT2. Interestingly, the use of type II GR agonist clearly produces an increase of ACTH at ZT10. Here the question arises about whether the effects of the agonists and antagonists can be due to diffusion of the microdialyzed drugs into the portal system and then to the pituitary to reach the corticotrophs. However, the increase in ACTH at ZT10 cannot be due to the action of GR agonist on the corticotrophs in the pituitary, because the agonist would inhibit ACTH release there (Deng et al., 2015). In the same sense, the action of antagonists on corticotrophs should increase the release of ACTH (Kling et al., 2008). This effect is not observed after type I GR antagonist administration at ZT2 or ZT10. Moreover, at ZT10, when the highest Cort values are achieved after antagonist is applied, we observed a drop of ACTH instead of the expected increase if the drug went to the portal system, which suggests that corticotrophs are not sensing the drug applied to the ARC, but rather respond to the actual increased Cort values. The changes in ACTH levels at ZT10 during dialysis with type II GR agonist and antagonist may be explained in part by the effects of glucocorticoid nongenomic action on the pituitary gland (Walker et al., 2012; Deng et al., 2015).

Previous experiments have demonstrated the action of both type I and type II GRs to regulate Cort production (Reul et al., 1987; Spencer et al., 1998; Atkinson et al., 2008); however, the site of action remained elusive. Here, we show that the ARC is importantly involved in the Cort feedback by a differential action of type I and type II GRs, dependent on the time and basal levels of Cort (Fig 4). At ZT2, when mostly type I GRs are occupied, an antagonist for this receptor and not for type II GRs in the ARC produced a fast and sustained increase in Cort levels, as previously reported for intravenous injections (Atkinson et al., 2008; Berardelli et al., 2010). On the other hand, at ZT10, when the Cort level is already high, the occupation of type II GR is more important to control GCs, as demonstrated by the increase in Cort levels after the infusion of type II GR antagonist. At this time, in contrast to ZT2, type I receptor antagonist is not enough to increase Cort production.

We observed a transient increase of Cort after antagonizing type I GRs at ZT10, during the circadian peak. This transient increase would indicate that the response of low-affinity type I GRs to high concentrations of Cort (Karst et al., 2005) to monitor the beginning of the surge of Cort at an early stage. Deferred effects may be mediated by type II GRs, as suggested by the later effects observed with type II antagonist at the same time point. In the same sense, our experiment with stress conditions shows that the type I GR agonist does not produce an immediate negative feedback at low Cort levels, suggesting that the occupation of low-affinity type I GRs may be necessary to initiate a counterregulatory effect.

In conclusion, the fast-feedback mechanism demonstrated in the present experiments suggests the existence of at least two mechanisms to control circulating Cort levels. First, a fast sensing of circulating Cort by the ARC immediately gives negative feedback on the secretion of Cort from the adrenal gland. Second, slow feedback mediated by hampered penetration of Cort into other brain areas is due to the presence of MDR1-type P-glycoprotein in the vasculature that slows down the transport of Cort in to the brain (Cordon-Cardo et al., 1989; Uhr et al., 2002). Consequently, areas such as the hippocampus and the bed nucleus of the stria terminalis are reached only by Cort many minutes after reaching the ARC. This mechanism may influence both the direct control of the adrenal glands as well as the control of ACTH secretion via their projections to the PVN (Johnson et al., 2016). Such a two-phase control system may initially prevent too high increases of Cort and may next allow longer-term modifications and setting of balance by other brain regions (Yi et al., 2006; Herrera-Moro Chao et al., 2016). It is attractive to propose similar mechanisms to control the circulating levels of these molecules.

## Synthesis

Reviewing Editor: Rae Silver, Columbia University Decisions are customarily a result of the Reviewing Editor and the peer reviewers coming together and discussing their recommendations until a consensus is reached. When revisions are invited, a fact-based synthesis statement explaining their decision and outlining what is needed to prepare a revision will be listed below. The following reviewer(s) agreed to reveal their identity: Russell Romeo, Stafford Lightman

The authors have gone to great lengths to respond to the reviewer's comments, and the new draft is markedly improved. It is the case however that confirmation of the results in a hypophysectomised animal will be necessary to totally convince people there is no indirect effect at the level of the pituitary. At this point, the data is compelling and deserves publication, but statements such as the last sentence in the abstract must be softened to reflect the need for confirmation of the results in a hypophysectomized animal.

Specifically in this sentence “Our findings show that Cort sensing in the ARC is essential to communicate blood Cort levels to adapt Cort secretion depending on circadian and stress conditions.”

-“..is essential could be changed to “..may be essential”

- There is something unclear as written. What does the second half of the sentence mean. Please rewrite.

Please update the excessively strong claim, correct the sentence, and upload the manuscript.
